# Primary Amenorrhea Revealing Adamantinomatous Craniopharyngioma Associated With Central Hypogonadotropic Hypogonadism in an Adolescent Girl: A Case Report

**DOI:** 10.7759/cureus.116323

**Published:** 2026-09-15

**Authors:** Anshika Shanker, Gaurav Kannaujiya, Ravi K Sharma, Jashnoor Singh, Mohd Asad, Prashant Saini, Waqas Alauddin

**Affiliations:** 1 Medicine, Naraina Medical College and Research Centre, Kanpur, IND; 2 Medicine, Government Institute of Medical Sciences, Greater Noida, IND; 3 Medicine, T.S. Misra Medical College and Hospital, Lucknow, IND; 4 Physiology, Venkateswara Institute of Medical Sciences, Amroha, IND

**Keywords:** adamantinomatous craniopharyngioma, central hypogonadotropic hypogonadism, delayed puberty, hypothalamic-pituitary dysfunction, primary amenorrhea, suprasellar tumor

## Abstract

Adamantinomatous craniopharyngioma is an uncommon benign epithelial tumor arising from remnants of Rathke's pouch within the sellar-suprasellar region. Although patients typically present with headache, visual impairment, growth retardation, or multiple endocrine abnormalities, primary amenorrhea as the initial clinical presentation is unusual, particularly in the absence of neurological symptoms. We report the case of a 15-year-old adolescent girl who presented with primary amenorrhea and delayed pubertal development without headache, visual complaints, or other focal neurological deficits. Endocrine evaluation revealed central hypogonadotropic hypogonadism, raising suspicion of hypothalamic-pituitary axis dysfunction. Magnetic resonance imaging of the brain demonstrated a well-defined multilobulated, predominantly cystic sellar-suprasellar lesion measuring approximately 2.1 cm × 2.2 cm × 2.3 cm, containing a small enhancing solid component and imaging features suggestive of calcification. The lesion extended superiorly to the floor of the third ventricle and compressed the optic chiasm, with imaging findings highly suggestive of adamantinomatous craniopharyngioma. The patient was initially evaluated in the gynecology department and was subsequently referred to pediatric endocrinology. Following endocrine and radiological evaluation, multidisciplinary management involving pediatric endocrinology and neurosurgery was undertaken. This case highlights the importance of considering hypothalamic-pituitary lesions in adolescents presenting with primary amenorrhea and central hypogonadotropic hypogonadism, even in the absence of neurological manifestations. Early endocrine evaluation followed by timely neuroimaging is crucial for establishing the diagnosis, preventing irreversible visual and hypothalamic complications, and facilitating appropriate multidisciplinary management. This report emphasizes that primary amenorrhea may represent the earliest manifestation of adamantinomatous craniopharyngioma and underscores the importance of maintaining a high index of suspicion in adolescents with unexplained delayed puberty.

## Introduction

Craniopharyngiomas are rare benign epithelial tumors arising from embryonic remnants of Rathke's pouch and account for approximately 2%-5% of all primary intracranial neoplasms [1,2]. These tumors are predominantly located within the sellar and suprasellar region and are classified as World Health Organization (WHO) grade 1 lesions [1,2]. Despite their benign histopathological nature, craniopharyngiomas often demonstrate locally invasive behavior because of their proximity to critical structures, including the optic chiasm, hypothalamus, pituitary gland, and third ventricle. Consequently, patients may develop significant neurological, visual, and endocrine complications that substantially affect long-term quality of life [1,2].

Craniopharyngiomas are classified into two distinct histopathological subtypes: adamantinomatous and papillary. The adamantinomatous subtype is the predominant variant in children and adolescents and is characterized by cyst formation, calcification, peripheral palisading epithelium, wet keratin, and activating mutations of the CTNNB1 gene [2,3]. In contrast, papillary craniopharyngioma occurs almost exclusively in adults and is strongly associated with BRAF V600E mutations [2,3].

The clinical presentation of craniopharyngioma is heterogeneous and depends on the tumor's size, growth pattern, and involvement of adjacent neuroendocrine structures. Common manifestations include headache, visual disturbances, growth retardation, delayed puberty, and pituitary hormone deficiencies resulting from hypothalamic-pituitary dysfunction [1,4]. Although endocrine abnormalities are frequently observed, primary amenorrhea as the initial clinical presentation is uncommon, particularly in adolescents who lack overt neurological or visual symptoms. Such atypical presentations may delay diagnosis and postpone definitive treatment, thereby increasing the risk of irreversible endocrine and visual sequelae [5].

Primary amenorrhea associated with central hypogonadotropic hypogonadism should prompt a systematic evaluation of the hypothalamic-pituitary axis after excluding chromosomal abnormalities, gonadal dysgenesis, and congenital reproductive tract anomalies. In this setting, magnetic resonance imaging (MRI) of the brain is essential for identifying structural lesions involving the sellar and suprasellar region and establishing the underlying etiology [6].

We report the case of a 15-year-old girl who presented with primary amenorrhea as the initial manifestation of adamantinomatous craniopharyngioma associated with central hypogonadotropic hypogonadism due to hypothalamic-pituitary dysfunction. This case highlights the importance of considering intracranial sellar-suprasellar lesions in the differential diagnosis of adolescents presenting with unexplained primary amenorrhea and delayed puberty. It further emphasizes the critical role of early endocrine assessment and timely neuroimaging in facilitating prompt diagnosis, appropriate multidisciplinary management, and prevention of long-term neurological and endocrine complications.

## Case presentation

A 15-year-old girl initially presented to the gynecology outpatient department with primary amenorrhea. Following an initial gynecological assessment, she was referred to the endocrinology department for further evaluation of delayed pubertal development and a possible underlying endocrine disorder. She had not attained menarche despite being of appropriate chronological age. There was no history of cyclical lower abdominal pain, headache, vomiting, seizures, visual disturbances, galactorrhea, polyuria, polydipsia, excessive thirst, or significant weight loss. Her antenatal, birth, and developmental histories were unremarkable. There was no family history of delayed puberty, primary amenorrhea, endocrine disorders, or intracranial neoplasms.

On physical examination, the patient was hemodynamically stable. Clinical examination revealed delayed pubertal development with underdeveloped secondary sexual characteristics. Pubertal assessment revealed Tanner stage I breast development, with Tanner stage I pubic and axillary hair, consistent with markedly delayed development of secondary sexual characteristics. Her height was 145 cm, weight was 59 kg, and BMI was 28.1 kg/m². No dysmorphic features suggestive of chromosomal abnormalities were identified. Neurological examination, including cranial nerve assessment, was normal, and no focal motor or sensory deficits were detected. Bedside visual acuity and confrontation visual field testing did not demonstrate any apparent visual impairment.

Written informed consent for publication was obtained from the patient's parent/legal guardian, and informed assent was obtained from the patient. Consent included permission to publish the clinical information and accompanying images related to this case report.

The patient was initially evaluated in the gynecology outpatient department for primary amenorrhea. A detailed gynecological history was obtained, followed by clinical examination and evaluation for anatomical and developmental causes of primary amenorrhea. Pelvic ultrasonography revealed a uterus that was small for the patient’s age, measuring approximately 3.7 cm × 9.1 cm. The right ovary measured approximately 2.1 cc, with no obvious dominant follicle, whereas the left ovary was not adequately visualized. Karyotyping demonstrated a normal female chromosomal complement (46,XX). No structural or chromosomal abnormality was identified to account for the primary amenorrhea. In view of delayed pubertal development, she was referred from the gynecology outpatient department to the endocrinology department for further evaluation.

At endocrinology evaluation, a comprehensive assessment of the hypothalamic-pituitary-gonadal axis was performed. Laboratory investigations demonstrated markedly suppressed serum follicle-stimulating hormone (FSH) and luteinizing hormone (LH) concentrations, consistent with central hypogonadotropic hypogonadism. Serum prolactin was within the normal reference range. Thyroid function tests, including thyroid-stimulating hormone (TSH), total triiodothyronine (T3), and total thyroxine (T4), were within normal limits, while morning serum cortisol was preserved. These findings supported the diagnosis of central hypogonadotropic hypogonadism and raised suspicion of hypothalamic-pituitary dysfunction, prompting further radiological evaluation with MRI of the brain.

Given the biochemical evidence of central endocrine dysfunction, contrast-enhanced MRI of the brain with a dedicated sellar protocol was performed. MRI demonstrated a well-defined, multilobulated, predominantly cystic lesion centered within the sellar-suprasellar region, measuring approximately 2.1 cm × 2.2 cm × 2.3 cm (Figures 1, 2).

**Figure 1:**
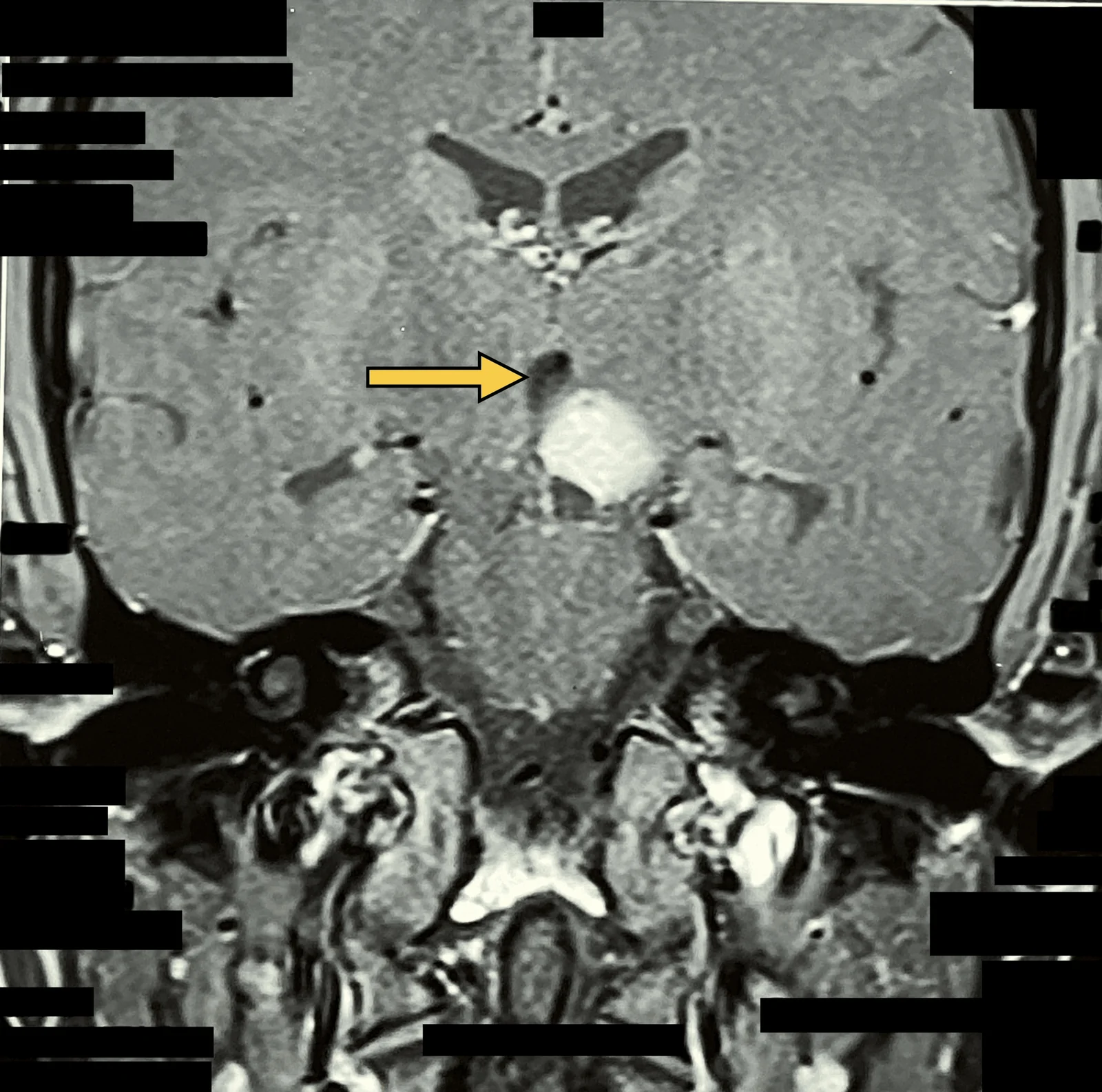
Coronal contrast-enhanced magnetic resonance image demonstrating the suprasellar lesion Coronal post-contrast T1-weighted magnetic resonance image demonstrating a well-defined, predominantly cystic sellar-suprasellar lesion (yellow arrow) with superior extension toward the floor of the third ventricle. The imaging characteristics are suggestive of an adamantinomatous craniopharyngioma.

**Figure 2:**
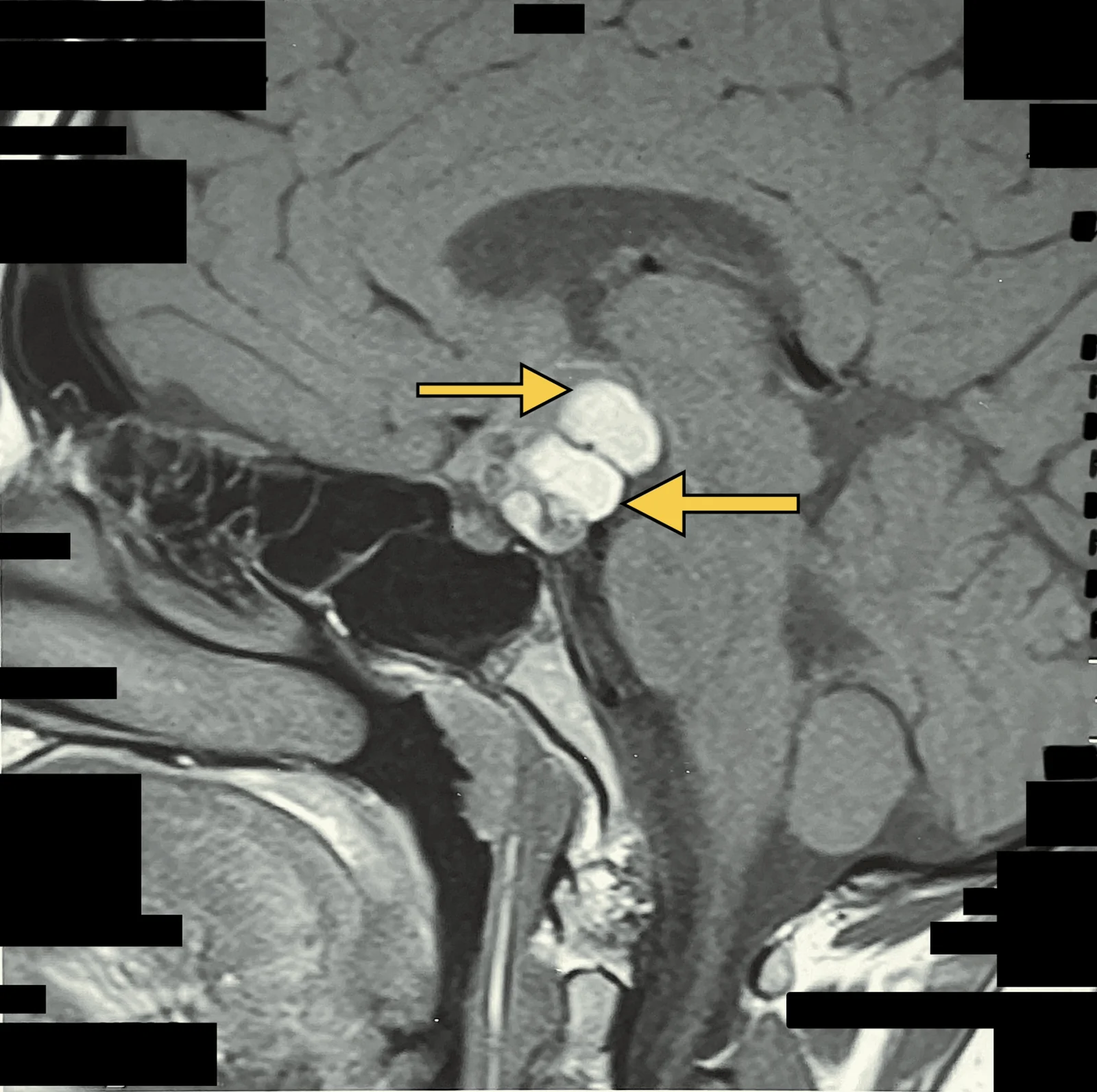
Sagittal magnetic resonance image demonstrating the multilobulated cystic morphology Mid-sagittal magnetic resonance image demonstrating a multilobulated, predominantly cystic sellar-suprasellar mass (yellow arrows) with a small solid component. The lesion extends superiorly toward the hypothalamic region and the floor of the third ventricle, consistent with the characteristic radiological appearance of adamantinomatous craniopharyngioma.

The lesion exhibited heterogeneous signal intensity with multiple cystic locules and a small enhancing solid component (Figure 2). Intralesional susceptibility artifacts were noted, suggesting the presence of calcification. Superiorly, the lesion extended to the floor of the third ventricle, while inferiorly it remained closely related to the pituitary gland.

The mass closely abutted the optic chiasm, hypothalamic region, cavernous sinus, and adjacent intracranial vessels without evidence of vascular encasement. Diffusion-weighted imaging did not demonstrate restricted diffusion. Coronal T2-weighted imaging further demonstrated superior displacement and compression of the optic chiasm by the suprasellar lesion (Figure 3). Overall, the imaging findings were highly suggestive of adamantinomatous craniopharyngioma.

**Figure 3:**
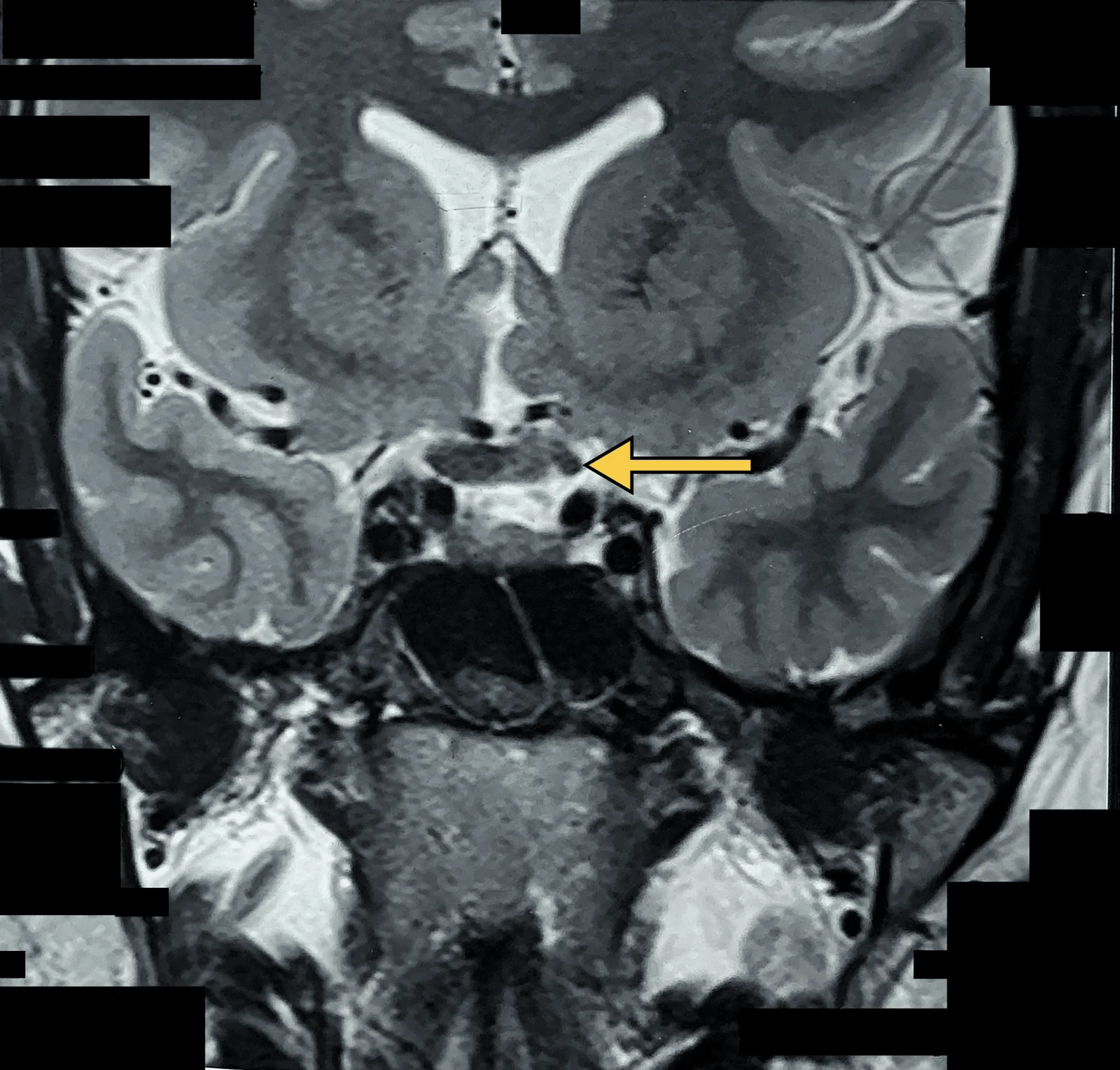
Coronal T2-weighted magnetic resonance image demonstrating optic chiasm compression Coronal T2-weighted magnetic resonance image demonstrating the suprasellar cystic lesion with compression and superior displacement of the optic chiasm (yellow arrow). The lesion occupies the sellar-suprasellar region and exerts mass effect on the visual pathway, a characteristic feature of adamantinomatous craniopharyngioma.

The patient’s clinical presentation (Table 1), biochemical evidence of central hypogonadotropic hypogonadism (Table 2), and characteristic MRI findings (Table 3) established a strong preoperative suspicion of adamantinomatous craniopharyngioma presenting with primary amenorrhea secondary to hypothalamic-pituitary dysfunction. Following multidisciplinary evaluation involving pediatric endocrinology and neurosurgery, the patient underwent surgical resection of the sellar-suprasellar lesion. Tumor tissue obtained during the surgical procedure was submitted for histopathological examination, which confirmed the diagnosis of adamantinomatous craniopharyngioma, CNS WHO grade 1. Microscopic evaluation demonstrated a solid-cystic lesion lined by squamous epithelium, with nests of squamous cells bordered by palisading columnar epithelium. Characteristic histopathological features, including wet keratin, stellate reticulum, dystrophic calcification, cholesterol clefts, and xanthogranulomatous reaction, were identified. Reactive gliosis and cystic degeneration were also observed. Following surgery, the patient was managed for pituitary hormone deficiencies and started on estrogen replacement therapy with Progynova for induction of pubertal development. She was advised long-term endocrine follow-up with periodic assessment of pituitary function and hormone replacement therapy as clinically indicated. Attainment of menarche was not documented during the available follow-up period.

**Table 1:** Clinical characteristics at presentation

Characteristic	Finding
Age	15 years
Sex	Female
Chief complaint	Primary amenorrhea
Headache	Absent
Visual symptoms	Absent
Seizures	Absent
Polyuria/polydipsia	Absent
Neurological examination	No focal neurological deficits
Initial clinical impression	Delayed puberty with suspected hypothalamic-pituitary pathology

**Table 2:** Baseline endocrine evaluation The markedly suppressed gonadotropin levels were consistent with central hypogonadotropic hypogonadism, suggesting hypothalamic-pituitary axis dysfunction and warranting further neuroimaging. FSH, follicle-stimulating hormone; LH, luteinizing hormone; TSH, thyroid-stimulating hormone; T3, triiodothyronine; T4, thyroxine

Investigation	Result	Reference range	Interpretation
FSH	0.3 mIU/mL	1.5-12.4	Markedly decreased
LH	0.3 mIU/mL	1.7-8.6	Markedly decreased
Prolactin	15.0 ng/mL	2.1-18.4	Within normal limits
Morning cortisol	13.9 µg/dL	6.2-19.4	Within normal limits
TSH	3.21 µIU/mL	0.27-4.20	Within normal limits
Total T3	174 ng/dL	91-218	Within normal limits
Total T4	11.0 µg/dL	5.9-13.2	Within normal limits

**Table 3:** MRI characteristics of the lesion MRI, magnetic resonance imaging

Imaging characteristic	Finding
Imaging modality	Contrast-enhanced brain MRI with dedicated sellar protocol
Location	Sellar-suprasellar region
Maximum dimensions	Approximately 2.1 cm × 2.2 cm × 2.3 cm
Morphology	Well-defined, multilobulated, predominantly cystic lesion
Solid component	Present
Calcification	Suggested by susceptibility (blooming) foci
Contrast enhancement	Mild heterogeneous enhancement of the solid component
Diffusion restriction	Absent
Third ventricular extension	Present
Optic chiasm	Superior displacement and compression
Pituitary gland	Closely abutting the lesion
Overall radiological impression	Imaging features highly suggestive of adamantinomatous craniopharyngioma

## Discussion

Craniopharyngiomas are rare benign epithelial tumors arising from embryonic remnants of Rathke's pouch and account for approximately 2%-5% of primary intracranial neoplasms. Despite their benign histology (WHO grade 1), these tumors often cause significant neurological, visual, and endocrine morbidity because of their close relationship with the hypothalamus, pituitary gland, optic chiasm, and third ventricle [1,2]. The adamantinomatous subtype predominates in children and adolescents and is characterized by cyst formation, calcification, wet keratin, and CTNNB1 (β-catenin) mutations, whereas the papillary subtype is more common in adults and is associated with BRAF V600E mutations [2,3].

The clinical presentation varies according to tumor size and involvement of adjacent structures. Headache, visual disturbances, growth retardation, delayed puberty, and pituitary hormone deficiencies are the most common manifestations in pediatric patients [4,5]. However, primary amenorrhea as the initial symptom is uncommon, particularly in adolescents without neurological or visual symptoms. Consequently, the diagnosis may be overlooked because the initial evaluation is often focused on gynecological or endocrine causes.

Primary amenorrhea associated with central hypogonadotropic hypogonadism should prompt evaluation of the hypothalamic-pituitary axis after exclusion of chromosomal, gonadal, and Müllerian abnormalities. Markedly reduced FSH and LH concentrations should prompt dedicated MRI of the sellar-suprasellar region to identify structural lesions [6-8]. In our patient, endocrine abnormalities preceded neurological manifestations, and MRI subsequently demonstrated a multilobulated, predominantly cystic suprasellar lesion with imaging features characteristic of adamantinomatous craniopharyngioma. This case illustrates that endocrine dysfunction may be the earliest clinical manifestation of an otherwise clinically silent intracranial tumor.

MRI is the imaging modality of choice for evaluating sellar-suprasellar masses. Adamantinomatous craniopharyngiomas typically appear as well-defined, multiloculated, predominantly cystic lesions with variable solid components and heterogeneous signal intensity [7-9]. Calcification, although best demonstrated on computed tomography, may also be suggested on MRI by susceptibility or blooming artifacts [9,10]. In our patient, the combination of a predominantly cystic multilobulated lesion, a small enhancing solid component, superior extension to the floor of the third ventricle, and optic chiasm compression strongly supported the diagnosis. The principal differential diagnoses include Rathke's cleft cyst, cystic pituitary adenoma, germinoma, hypothalamic glioma, and arachnoid cyst [9,10].

Although endocrine dysfunction is a well-recognized feature of craniopharyngioma, primary amenorrhea as the initial presentation has been reported infrequently. Most published pediatric cases have presented with headache, visual impairment, growth failure, or established hypopituitarism before diagnosis [4,5]. In contrast, our patient presented primarily with primary amenorrhea and central hypogonadotropic hypogonadism, without clinically significant neurological or ophthalmological symptoms. This emphasizes the importance of considering intracranial pathology in adolescents with unexplained delayed puberty or primary amenorrhea.

Management requires a multidisciplinary approach involving endocrinologists, neurosurgeons, neuroradiologists, and ophthalmologists. Current treatment strategies favor maximal safe surgical resection, with adjuvant radiotherapy reserved for residual or recurrent disease to optimize long-term tumor control while minimizing hypothalamic and pituitary injury [11,12]. Lifelong endocrine surveillance is essential because pituitary hormone deficiencies and tumor recurrence may occur despite successful treatment.

This case highlights three important clinical messages. First, primary amenorrhea may be the earliest manifestation of an otherwise asymptomatic sellar-suprasellar tumor. Second, adolescents with central hypogonadotropic hypogonadism should undergo timely hypothalamic-pituitary imaging to exclude structural intracranial lesions. Finally, early diagnosis through coordinated endocrine evaluation and MRI facilitates prompt multidisciplinary management and may prevent irreversible neurological, visual, and endocrine complications.

## Conclusions

Primary amenorrhea may represent the earliest clinical manifestation of adamantinomatous craniopharyngioma. Adolescents presenting with unexplained primary amenorrhea and central hypogonadotropic hypogonadism should undergo prompt evaluation of the hypothalamic-pituitary axis, including dedicated sellar MRI, even in the absence of neurological or visual symptoms. Early recognition and multidisciplinary management are essential to optimize endocrine outcomes and reduce the risk of long-term neurological and visual complications.
